# Patterns of Diversity of the Rissoidae (Mollusca: Gastropoda) in the Atlantic and the Mediterranean Region

**DOI:** 10.1100/2012/164890

**Published:** 2012-05-22

**Authors:** Sérgio P. Ávila, Jeroen Goud, António M. de Frias Martins

**Affiliations:** ^1^Departamento de Biologia, Universidade dos Açores, 9501-801 Ponta Delgada, Açores, Portugal; ^2^CIBIO-Açores, Universidade dos Açores, 9501-801 Ponta Delgada, Açores, Portugal; ^3^MPB-Marine PalaeoBiogeography Working Group of the University of the Azores, Rua da Mãe de Deus, 9501-801 Ponta Delgada, Açores, Portugal; ^4^National Museum of Natural History, Invertebrates, Naturalis Darwinweg, Leiden, P.O. Box 9517, 2300 RA Leiden, The Netherlands

## Abstract

The geographical distribution of the Rissoidae in the Atlantic Ocean and Mediterranean Sea was compiled and is up-to-date until July 2011. All species were classified according to their mode of larval development (planktotrophic and nonplanktotrophic), and bathymetrical zonation (shallow species—those living between the intertidal and 50 m depth, and deep species—those usually living below 50 m depth). 542 species of Rissoidae are presently reported to the Atlantic Ocean and the Mediterranean Sea, belonging to 33 genera. The Mediterranean Sea is the most diverse site, followed by Canary Islands, Caribbean, Portugal, and Cape Verde. The Mediterranean and Cape Verde Islands are the sites with higher numbers of endemic species, with predominance of *Alvania* spp. in the first site, and of *Alvania* and *Schwartziella* at Cape Verde. In spite of the large number of rissoids at Madeira archipelago, a large number of species are shared with Canaries, Selvagens, and the Azores, thus only about 8% are endemic to the Madeira archipelago. Most of the 542-rissoid species that live in the Atlantic and in the Mediterranean are shallow species (323), 110 are considered as deep species, and 23 species are reported in both shallow and deep waters. There is a predominance of nonplanktotrophs in islands, seamounts, and at high and medium latitudes. This pattern is particularly evident in the genera *Crisilla, Manzonia, Onoba, Porosalvania, Schwartziella*, and *Setia*. Planktotrophic species are more abundant in the eastern Atlantic and in the Mediterranean Sea. The results of the analysis of the probable directions of faunal flows support the patterns found by both the Parsimony Analysis of Endemicity and the geographical distribution. Four main source areas for rissoids emerge: Mediterranean, Caribbean, Canaries/Madeira archipelagos, and the Cape Verde archipelago. We must stress the high percentage of endemics that occurs in the isolated islands of Saint Helena, Tristan da Cunha, Cape Verde archipelago and also the Azores, thus reinforcing the legislative protective actions that the local governments have implemented in these islands during the recent years.

## 1. Introduction

Rapoport's latitudinal rule relates geographical distribution with latitude [1, 2]. This rule states that the range of the geographical distribution of species increases with latitude [3]. Several hypotheses were provided: the seasonal variability hypothesis [2, 4], the differential extinction hypothesis [3], the competition hypothesis [5–7], or the Milankovitch climate oscillations, which force larger distributional changes [8].

Although studies relating biological diversity with latitude usually use higher taxonomical categories, recent papers restricted to checklists of marine molluscs have been used to address this issue [9–12]. The papers of Roy et al. [9, 10], who used lists with 3,916 marine Caenogastropod species geographically distributed along the north and Central America shores between 10°S–83°N (west-Atlantic shores: 2,009 species; east-Pacific shores: 1,907 species), confirmed the latitudinal gradient pattern, with the number of species decreasing with latitude. However, they did not confirm the seasonal variability hypothesis, as the mean geographical range decreased from the tropics to higher latitudes (see [9], Figure  3). These authors concluded that the energy hypothesis [13, 14], that correlates the surface incident solar radiation and its correspondent average sea surface temperature (SST) with latitude and number of species, better explained this biogeographical pattern. This relationship is higher outside the tropical latitudes [9]. Notwithstanding solar radiation is a simple function of the latitude, SSTs are a complex function of climatic variables, oceanic currents, and other factors (e.g., local submarine topography, upwelling, estuarine systems with high discharge of both nutrients and sediments, etc.) [10, 15, 16]. 

The Rissoidae are a family of small-sized, marine to brackish-water gastropod molluscs. This very diverse family was taxonomically reviewed by Wenz [17], Coan [18], Nordsieck [19] and Ponder [20, 21]. Two subfamilies are presently recognized: Rissoinae and Rissoininae [22]. Given its species-abundance, easy preservation in fossilized form, and the fact that key-elements of the life cycle can be obtained from shell morphology, rissoids have a large potential for evolutionary studies [23]. 

Published information about the Atlantic and Mediterranean Rissoidae is vast and is scattered among a wide variety of journals but, with a few exceptions, these studies are typically geographically localized. Many species descriptions are usually based on shell morphology and on only a few specimens, most of them dead shells. At present, there is a lack of a background scenario of the geographical distribution for this family in the Atlantic and in the Mediterranean. No phylogeny has been established for this family.

To our knowledge, this is the first attempt to summarize present information about the geographic distributional pattern of this family in the Atlantic Ocean and the Mediterranean Sea, with the purpose of identifying the biotic similarities between areas. 

## 2. Materials and Methods

### 2.1. Geographical Distribution

 The geographical distribution of the Rissoidae in the Atlantic Ocean and Mediterranean Sea was compiled through an exhaustive search of the primary literature and is up to date until July 2011. The following sites and references were considered:

- ARC: Arctic: above 75° N: Warén [24, 25], Hansson [26],
- GRE: Greenland, western shores of Baffin Island, Baffin Bay, Davis Strait, and Labrador Sea: Bouchet and Warén [27], Hansson [26],
- ICE: Iceland: Warén [28, 29],
- SCA: Scandinavia: Norway Sea, Skagerrak and Kattegat, Baltic Sea and Faroe Islands: Fretter and Graham [30], Warén [24, 29], Hansson [26],
- BRI: British Isles: Smith [31], Fretter and Graham [30], Killeen and Light [32],
- POR: western Atlantic Iberian façade (from Cabo Vilán, western Galician shores, down to Cape São Vicente) and southern shores of Algarve, Portugal): Nobre [33, 34], Nobre and Braga [35], Macedo et al. [36], Rolán [37],
- MED: Mediterranean: Nordsieck [19], Aartsen and Fehr-de-Wal [38], Aartsen [39–47], Verduin [48–50], Aartsen and Verduin [23, 51], Palazzi [52], Aartsen et al. [53], Amati [54–56], Amati and Nofroni [57–59], Amati and Oliverio [56], Oliverio [60–62], Oliverio et al. [63], Aartsen and Linden [64], Linden and Wagner [65], Hoenselaar and Moolenbeek [66], Aartsen and Menkhorst [53], Giusti and Nofroni [67], Aartsen et al. [68], Amati et al [69], Hoenselaar and Hoenselaar [70], Nofroni and Pizzini [71], Oliverio et al. [72], Aartsen and Engl [73], Smriglio and Mariottini [74], Margelli [75], Bogi and Galil [76], Buzzurro and Landini [77], Peñas et al. [78], Oliver and Templado [79], CIESM, CLEMAM,

- AZO: Azores: Watson [80], Dautzenberg [81], Amati [82], Gofas [83], Oliverio et al. [72], Linden [84], Linden and van Aartsen [85], Ávila [86–88],
- LUS: Lusitanian group of seamounts (a chain of seamounts located between Portugal and Madeira): Gorringe, Josephine, Ampère, Seine: Ávila and Malaquias [89], Beck et al. [90], Gofas [91],
- MET: Meteor group of seamounts (located about 600 km south of the Azores): Great Meteor, Irving, Atlantis, Hyères, Plato, Tyro, Cruiser: Gofas [91],
- MAD: Madeira, Porto Santo and Desertas Islands, Nobre [92], van Aartsen [47], Palazzi [52], Verduin [93], Moolenbeek and Hoenselaar [94, 95], Segers et al. [96],
- SEL: Selvagens Islands: Verduin [93], Amati [97],
- CAN: Canary Islands: van Aartsen [47], Moolenbeek and Faber [98], Rolán [99], Verduin [93], Linden and Wagner [100], Moolenbeek and Hoenselaar [94, 95, 101], Segers [102], Hernández-Otero et al. [103],
- CAP: Cape Verde archipelago: Rolán [104], Moolenbeek and Rolán [105], Templado and Rolán [106], Rolán and Rubio [107], Rolán and Luque [108], Rolán [109], Rolán and Oliveira [110],
- STH: Saint Helena Island: Smith [111], MALACOLOG,
- TRS: Tristão da Cunha Island: Worsfold et al. [112], MALACOLOG,
- WAF: West African shores—Atlantic Morocco, from Straits of Gibraltar south, Western Sahara, and Mauritania, Cape Verde (Senegal): Verduin [49], Gofas and Warén [113], Moolenbeek and Piersma [114], Rolán and Fernandes [115], Gofas [116, 117],
- ANG: Angola: Rolán and Ryall [118], Rolán and Fernandes [115], Gofas [116],
- NSC: New Scotia biogeographical province—Atlantic shores of USA, between Newfoundland (50° N) and Cape Cod (42° N): MALACOLOG,
- VIR: Virginian biogeographical province *sensu* Engle and Summers [119]—Atlantic shores of USA, between Cape Cod (42° N) and Cape Hatteras, North Carolina (35° N): MALACOLOG,
- CRL: Carolinian biogeographical province–Atlantic shores of USA, between Cape Hatteras, North Carolina (35° N) and Cape Canaveral (28°30′ N): Rex et al. [120], MALACOLOG,

- TRO: Tropical biogeographical province (from now on generically designated as “Caribbean”)—Atlantic shores of USA, south of Cape Canaveral (28°30′ N), including western and eastern shores of Florida, Gulf of Mexico (Louisiana and Texas shores, as well as Yucatan Peninsula, México), Bahamas, Caribbean Sea, south to Cabo Frio (Brazil) (23°S): Dall [121], Baker et al. [122], Faber and Moolenbeek [123], Jong and Coomans [124], Leal and Moore [125], Faber [126], Leal [127], Rolán [128], Espinosa and Ortea [129], Rosenberg et al. [130] MALACOLOG,
- BRA: Biogeographical province of Brazil (this includes the Paulista and Patagonic Provinces *sensu* Palacio [131])—from Cabo Frio (23°S) south to River Plate (35°S): MALACOLOG,
- SSA: southeast of South America—biogeographical province of Malvinas (*sensu* Palacio [131]) Atlantic shores from River Plate (35°S) south to Tierra del Fuego and Cape Horn, including Los Estados Island, Falkland Islands (Malvinas), Burdwood Bank and South Georgia Island: Ponder [132], Ponder and Worsfold [133],
- ANT: Antarctic—from 60°S south, including South Orkney Islands (Signy Island), South Shetland islands, Antarctic Peninsula and Weddell Sea: Ponder [132].

We have also consulted other bibliographical sources, with a wider systematical or geographical subject, such as Babio and Thiriot-Quiévreux [134], Aartsen [40], Fretter and Graham [30], Ponder [21], Verduin [135], Templado and Rolán [136], Hoenselaar and Moolenbeek [66], Moolenbeek and Hoenselaar [137], Moolenbeek and Faber [138–140], Sleurs [141–143], Hoenselaar [70, 144], Bouchet and Warén [27], Sleurs and Preece [145], Warén [146], Hoenselaar and Goud [147], Goud [148], Gofas et al. [149], Rolán [150], Gofas [91], and Garilli [151]. MALACOLOG, CIESM, CLEMAM, and WoRMS web databases were very useful and widely consulted. 

### 2.2. Bathymetrical Zonation

The bathymetrical zonation considers shallow species (those living between the intertidal and 50 m depth) and deep species (those usually living below 50 m depth). The choice of the threshold at 50 m depth is related with the following reasons: (i) algal species to which Rissoidae are very often associated are rare below 50 m depth; (ii) direct sampling by scuba-diving is more frequent in waters less than 50 m depth; (iii) in waters deeper than 50 m depth, usually the samplings are obtained via indirect methodologies (grabs, most often).

The complete database was last updated in October 2011 and is available from the authors upon request. 

### 2.3. Modes of Larval Development

All species were classified according to their mode of larval development and bathymetrical zonation. Rissoids lay ovigerous capsules in the substrate that originate larvae with different modes of larval development. The extension of the larval phase reflects on the capabilities of dispersal and this has important ecological and historical biogeographical implications, related with the geographical distribution of the species. Two types of larval development were considered: planktotrophic (with a free-swimming feeding stage) and nonplanktotrophic (either lecithotrophic or direct development, both without a free-swimming feeding stage) [21, 89, 152, 153]. As almost nothing is known about the life cycle of rissoids, the mode of larval development was determined indirectly, through the analysis of the protoconch. In the Rissoidae family, multispiral protoconchs, with a small nucleus (usually less than 200 *μ*m) and with several whorls (typically more than 2), are associated with a planktotrophic mode of larval development. In most of the planktotrophic species, it is possible to discern between protoconch I and protoconch II. This is especially evident in species that possess a “sinusigera” larva, as is the case of *Alvania cancellata* [154]. Paucispiral protoconchs, with nucleus usually larger than 200 *μ*m, typically more than 300 *μ*m [87], and with about 1–1.5 whorls are related with a nonplanktotrophic mode of larval development [87, 88, 152, 155–157].

### 2.4. Biotic Similarities between Areas: Parsimony Analysis of Endemicity (PAE)

Rosen and Smith [158] developed the Parsimony Analysis of Endemicity or PAE method, which, under a cladistic framework, classifies areas in accordance with their shared taxa. This method is particularly useful when the researchers have no phylogenetic information to incorporate into the analysis, therefore, providing a primary description of the distributional pattern of the taxa and the biotic similarities between areas [159]. The original data resumes to a matrix with the presence/absence of the selected species in the studied areas. Thus, data for PAE consists of Area x Taxa matrices and the cladograms that result from this analysis represent nested sets of areas and correspond to the most parsimonious solution [160, 161]. The original Area x Taxa matrix with the geographical distribution of the Rissoidae was split in two, the first containing the shallow (<50 m depth), and the second with the deep (>50 m) rissoid species. Both shallow and deep rissoid species' matrices were analysed as follows.

Four species considered as Lessepsian and thus reported to the Mediterranean were removed from the initial database: *Alvania dorbignyi* [162], *Rissoina bertholleti*, *Rissoina spirata* (Sowerby, 1825), and *Voorwindia tiberiana* [163]. Unique taxa (restricted endemics, autapomorphies) were also removed prior to the PAE analysis [158]. No cosmopolitan taxa (plesiomorphies) were found in either of the matrices (shallow and deep rissoids). Low diversity localities (e.g., Brazil, Virginian Province, Tristan da Cunha Island, and Antarctic) were kept in the analysis. One outgroup with an all-0 score was added to the first row of the data matrix, to allow topologies to be rooted [160, 164]. Tree reconstruction was based on the heuristic search algorithm in PAUP* version 4.0 beta 10 [165] including 1,000 random stepwise-addition sequence replicates with tree-bisection-reconnection (TBR) and MULTREES on, and Acctran optimization in effect, but restricting the number of optimal trees per replicate to 1. Consensus trees (50% majority rule) were generated when more than 1 parsimonious tree resulted from the analysis, using 200 random addition sequence replicates with TBR and MULTREES on, and Acctran optimization in effect. One thousand bootstrap replicates with 100 random additions per replicate, with TBR and MULTREES on, and decay indices (“branch support” of Bremer [166]), measured support for individual nodes.

### 2.5. Biotic Similarities between Areas: Probable Directions of Faunal Flows

The analysis of the historical relationships between the selected areas was complemented by using the following formulas (*X*_*A*_ and *X*_*B*_) for each pair of areas (*A* and *B*) [167]:

(1) $$\begin{array}{c} X_{A}=\frac{\left(\text{number}\;\text{of}\;\text{species}\;\text{present}\;\text{in}\;\text{areas}\;A\;\text{and}\;B\right)}{A},\\ X_{B}=\frac{\left(\text{number}\;\text{of}\;\text{species}\;\text{present}\;\text{in}\;\text{areas}\;A\;\text{and}\;B\right)}{B}, \end{array}$$

where *A* is the total number of rissoid species present in area *A*, and *B* is the total number of rissoid species present in area *B*. When a faunal flow happened in historical times, from a source area to the target area, we expect the target area to show a subset of the species present in the source area. So, different values of the two indices (*X*_*A*_ and *X*_*B*_) are expectable, and the source area must have the smaller value [167].

## 3. Results

 Rissoidae family comprises 47 valid genera, some with a worldwide distribution (*Manzonia*, *Rissoina*, *Zebina*, *Stosicia*, *Pusillina*, and *Alvania*), while others are geographically restricted. According to Ponder [21], the genera *Attenuata*, *Lamellirissoina*, *Lironoba*, *Lucidestea*, *Merelina*, *Parashiela*, *Striatestea*, and *Voorwindia* are restricted to the Indo-Pacific. However, Leal [127] reported a species of *Lironoba* from Brazil (Tropical Province). Some genera solely occur in the Indian Ocean, for example, *Fenella* (Madagascar and Red Sea), while the genera *Tomlinella* is restricted to Reunion Island and Mauritius and the recently described *Porosalvania *is restricted to the Meteor group of seamounts [91]. 

### 3.1. Geographical Distribution

Five hundred and forty-two species of Rissoidae are presently reported to the Atlantic Ocean and the Mediterranean Sea, belonging to 33 genera. Six genera are represented by a single species: *Amphirissoa cyclostomoides* Dautzenberg and Fischer, [168], a nonplanktotrophic deep species that occurs in the Caribbean, in the Azores and in the Meteor group of seamounts; *Benthonella tenella* (Jeffreys [169], *B*. *gaza* Dall [170], and *B*. *fischeri* Dall, 1889 are synonyms), a widespread planktotrophic deep species; *Lironoba* sp., a nonplanktotrophic species restricted to the Atol das Rocas [127]; *Galeodinopsis tiberiana * (Coppi, 1876) (= *Alvania fariai* [115]), a shallow planktotrophic species reported to the Cape Verde archipelago, West Africa, and Angola; *Pontiturboella rufostrigata* [171], a species restricted to the Black Sea; *Voorwindia tiberiana* [163], a shallow planktotrophic *Lessepsian* species that occurs in the Mediterranean.

The Mediterranean Sea is the most diverse site, with 160 species of Rissoidae, followed by Canary Islands (89 species), Caribbean (77), Portugal (74), and Cape Verde (67). The lowest diversity sites are the Carolinian Province (18 species), Greenland (16), Arctic (13), Angola (11), New Scotia Biogeographical Province (10), Antarctic (8), Virginian Biogeographical Province, Tristan da Cunha Island, and Brazil (all with just 7 species, Table 1).

The genera *Alvania* (74 species), *Rissoa* (26), *Setia* (18), and *Pusillina* (11) are species-abundant in the Mediterranean and along the Portuguese shores. *Boreocingula* and *Frigidoalvania* (as the name indicates) are restricted to higher latitudes (Arctic, Greenland, Iceland, and Scandinavia). *Boreocingula* also occurs in the British Isles, and *Frigidoalvania* is reported to the Atlantic shores of North America (NSC and VIR). *Onoba* is a genus with high number of species at Iceland and Greenland (9 and 5, resp.), but the most diverse sites are in the South Atlantic: 22 species at southeast of South America and 6 species at Tristan da Cunha Island and Antarctic (Table 1).

*Benthonellania*, *Folinia*, *Microstelma*, *Rissoina*, and *Zebina* have a higher number of species in the Caribbean (Tropical Province). *Crisilla* and *Manzonia* are particularly species-diverse in the Macaronesian archipelagos, especially at Canary Islands, Selvagens and Madeira, Porto Santo, and Desertas Islands. *Manzonia* is a specious genus also at the Lusitanian group of seamounts. *Schwartziella* spp. is very abundant at Cape Verde archipelago (26 species) as well as in the Carolinian and Tropical Provinces, and at Saint Helena Islands (4, 9, and 5 species, resp.). *Porosalvania* is a newly described endemic genus to the Meteor group of seamounts where it radiated into a number of species (Table 1).

*Stosicia* and *Voorwindia* occur predominantly in the Indo-Pacific Region [21], with just two species reported to the Western Atlantic, *Stosicia aberrans* [172], and *Stosicia houbricki * [173] (= *Stosicia fernandezgarcesi* [129], all restricted to the Tropical Province; *Voorwindia tiberiana* [163] occurs in the Mediterranean, where this *Lessepsian* species is considered as alien (nonestablished, CIESM database). However, fossil species of *Stosicia* are known from the Lower Miocene of the Eastern Atlantic and the Mediterranean [173]. 

### 3.2. Endemic Species

The Mediterranean and Cape Verde Islands are the sites with higher numbers of endemic species (71 and 58, resp.), with predominance of *Alvania* (37) in the first site, and of *Schwartziella* (26) and *Alvania* (20) at Cape Verde. Caribbean also has a high number of endemisms (57 species), especially of the genera* Alvania* (19) and* Rissoina* (13). British Isles, Angola, New Scotia, and Virginian Provinces do not have endemic species, and Brazil, Greenland, and Scandinavia only possess a single endemic species (Table 2). However, if these figures are viewed in percentages, Saint Helena Island, Cape Verde, and Tristan da Cunha are the sites with higher percentages of rissoid endemisms (90.0%, 86.6%, and 85.7%, resp.). Other sites with high percentage values are the Meteor group of seamounts (76.9%), the Caribbean (74.0%), southeastern shores of South America (66.7%), the Azores (44.7%), and the Mediterranean (44.4%). Antarctic (37.5%), the Lusitanian group of seamounts (37.0%), the West-African shores (24.0%), and Canary Islands (19.1%) also have a significant amount of endemic rissoids (Table 2).

In spite of the large number of rissoids at Selvagens (38 species), a large number of species are shared with Canaries (30) and Madeira (27) (Table 3), thus only 3 species are endemic to these islands (7.9%). Similar percentages of rissoid endemics occur at Greenland (6.3%) and Scandinavia (3.3%). Iceland has 12.0% of endemisms (Table 2).

### 3.3. Bathymetrical Zonation

 Most of the 542-rissoid species that live in the Atlantic and in the Mediterranean are shallow species (329). One hundred and forty-six are considered as deep species, living in waters with more than 50 m depth, and 23 species are reported to both shallow and deep waters. It was not possible to establish the bathymetrical zonation of 44 rissoid species.

*Benthonella*, *Benthonellania*, *Frigidoalvania*, *Gofasia*, *Microstelma*, and *Pseudosetia* typically are deep species, whereas *Botryphallus*, *Crisilla*, *Manzonia*, *Peringiella*, *Pusillina*, *Rissoa*, *Rissoina*, *Rudolphosetia*, *Schwartziella*, *Setia*, and *Zebina* are mostly constituted by shallow species. Some of these genera (e.g., *Rissoa* and *Rissoina*) are exclusively littoral. In the eastern-Atlantic shores and at latitudes higher than 55° N (Arctic, Greenland, Iceland, and Scandinavia), *Alvania* genus is mostly made of deep species; in all the other sites, usually this genus is predominantly dominated by shallow species (Table 4).

### 3.4. Modes of Larval Development

It was possible to infer the mode of larval development of 450 out of the 542 rissoid species, with 375 nonplanktotrophic species, and 75 planktotrophic species (Table 5). There is a predominance of nonplanktotrophs in islands, seamounts, and at high and medium latitudes. This pattern is particularly evident in the genera *Crisilla*, *Manzonia*, *Onoba*, *Porosalvania*, *Schwartziella*, and *Setia*. Planktotrophic species are more abundant in the eastern Atlantic and in the Mediterranean Sea. The British Isles and Angola are the only sites with excess of planktotrophs in relation to nonplanktotrophic rissoids. *Rissoa* is a very diverse genus in the Mediterranean Sea and along the shores of Portugal, and most are planktotrophic species. In the Arctic, Greenland, southeastern South America, and Antarctic, all rissoid species are nonplanktotrophs (Table 5).

 When the bathymetrical zonation of the rissoid species is analyzed in combination with the modes of larval development, some patterns emerge:

- most of the shallow nonplanktotrophic species occur in the Mediterranean sea, Cape Verde, and Canary islands, as well as Portugal, the Azores, Madeira archipelago, Selvagens, west African shores, Caribbean, and southeastern South America; *Alvania*, *Manzonia*, *Rissoa*, *Schwartziella,* and *Setia* are diverse genera in the north Atlantic archipelagos (Azores, Madeira, Selvagens, Canaries, and Cape Verde) (Table 6);
- shallow planktotrophic rissoid species are much more diverse along the European Atlantic shores, the west-African shores, the Mediterranean, and the Caribbean than in the Atlantic islands, with the exception of Canaries (Table 7);
- Scandinavia, British Isles, Portugal, Angola, and the Carolinian Province are the only sites with higher numbers of shallow planktotrophic species relative to the number of shallow non-planktotrophs (cf. Tables 6 and 7);
- deep nonplanktotrophic rissoid species are more diverse in the North Atlantic than in the South Atlantic; for instance, there are four such species in the Arctic and no species at all in the Antarctic: these species are also more diverse in the eastern Atlantic than in the western Atlantic shores (Table 8);
- deep planktotrophic rissoids are restricted to 4 genera, *Alvania*, *Benthonella*, *Benthonellania*, and *Obtusella* (Table 9);
- *Benthonella tenella* [169], the sole representative of this genus in the studied area, is the rissoid species with wider geographical range in the Atlantic; other species with large geographical ranges are *Obtusella intersecta* [174] and *Alvania cimicoides* [175]; all of them are deep planktotrophic species, although *Obtusella intersecta *may also occur in the littoral.

### 3.5. Biotic Similarities between Areas: Parsimony Analysis of Endemicity

 We used PAE separately on the shallow and on the deep rissoid species. After removing all the endemic species (no cosmopolitan species were found), 115 shallow species and 41 deep species of rissoids were analysed with the PAE methodology, using PAUP*.

**Figure 1:**
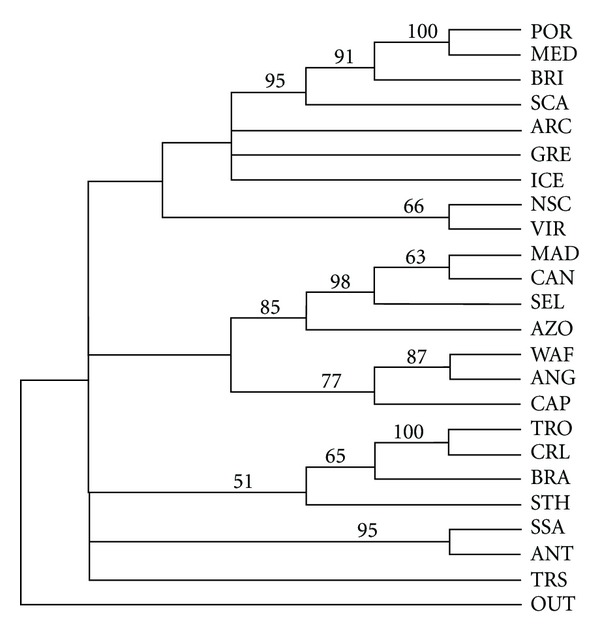
Consensus tree with bootstrap values for the shallow Rissoidae species.

 PAE of the shallow Atlantic and Mediterranean rissoids produced a single most parsimonious tree (*L* = 180, Ci = 0.6389, Ri = 0.7005) with three main groups. The first one strongly clusters Portugal, the Mediterranean, British Isles, and Scandinavia, with bootstrap values higher than 91%. A second group subdivides in two: the first subgroup, the Macaronesian archipelagos of Madeira, Canary Islands, Selvagens, and the Azores clusters; the second subgroup has West-African coast, Angola, and Cape Verde Islands. In a third group, western Atlantic sites are clustered: Caribbean and Carolinian Province cluster to Brazil at 65% bootstrap value. Saint Helena Island weakly clusters to the previous sites (bootstrap value of only 51%). New Scotia and Virginian Provinces cluster in an independent group (66%), as well as Southern South America and Antarctic (95%) (Figure 1).

**Figure 2:**
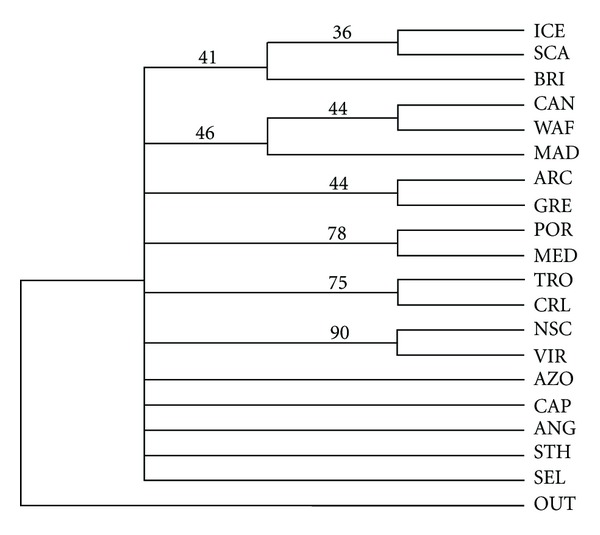
Consensus tree with bootstrap values for the deep Rissoidae species, abbreviations as in Table 1.

 The consensus tree (*L* = 82, Ci = 0.5000, Ri = 0.5816) that results from the PAUP* analysis of the deep rissoids is given in Figure 2. Bootstrap values are higher than 50% only for three groups: Portugal-Mediterranean (78%), Caribbean-Carolinian Province (75%), and New Scotia Province-Virginian Province. Some other sites also cluster, but at values lower than 50% (Figure 2).

### 3.6. Biotic Similarities between Areas: Probable Directions of Faunal Flows

**Figure 3:**
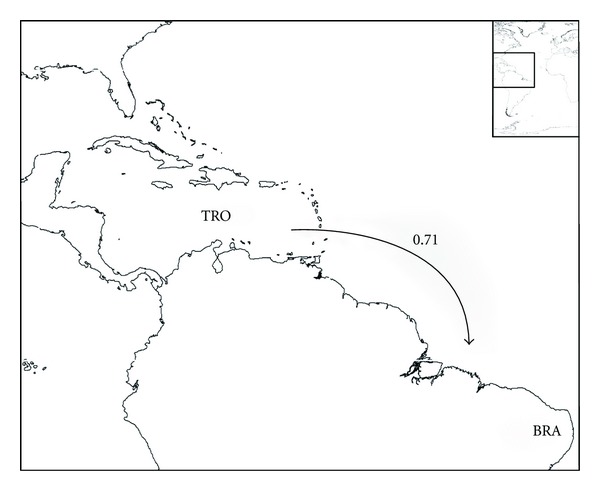
Probable colonization patterns of rissoid fauna in the central west-Atlantic. The arrows represent the probable main flux direction of faunas, and the associated numbers represent, for each pair of areas, the higher of the two similarity index values computed as described in the methods, abbreviations as in Table 1.

**Figure 4:**
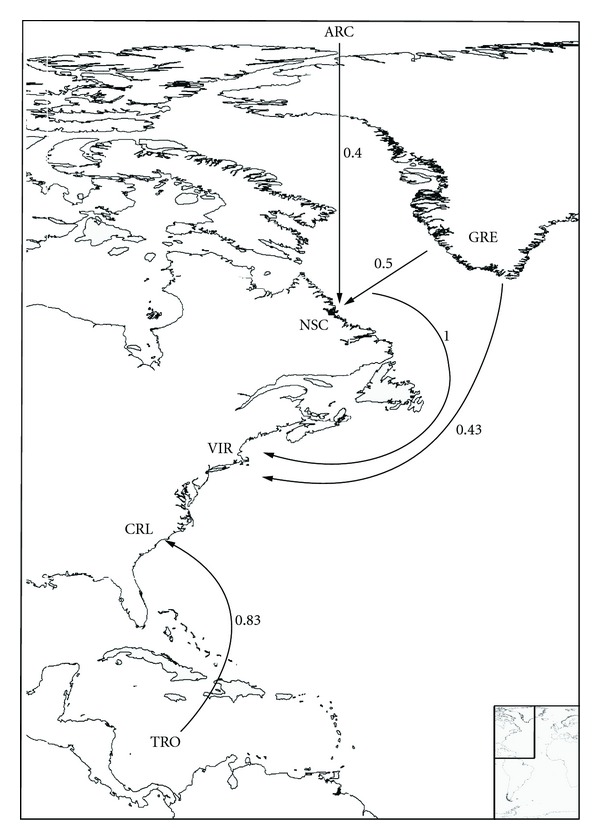
Probable colonization patterns of rissoid fauna in the Northwest Atlantic. The arrows represent the probable main flux direction of faunas, and the associated numbers represent, for each pair of areas, the higher of the two similarity index values computed as described in the methods, abbreviations as in Table 1.

**Figure 5:**
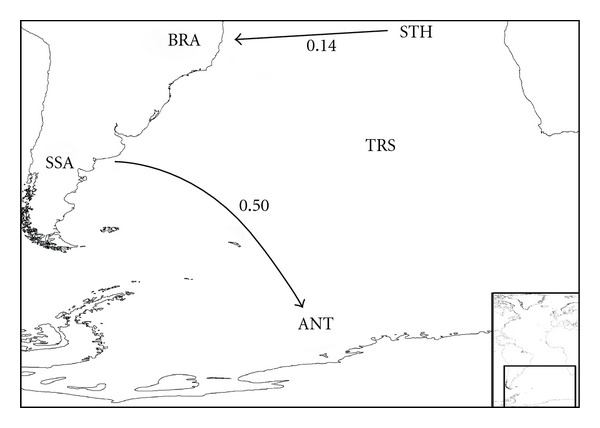
Probable colonization patterns of rissoid fauna in the South Atlantic. The arrows represent the probable main flux direction of faunas, and the associated numbers represent, for each pair of areas, the higher of the two similarity index values computed as described in the methods, abbreviations as in Table 1.

**Figure 6:**
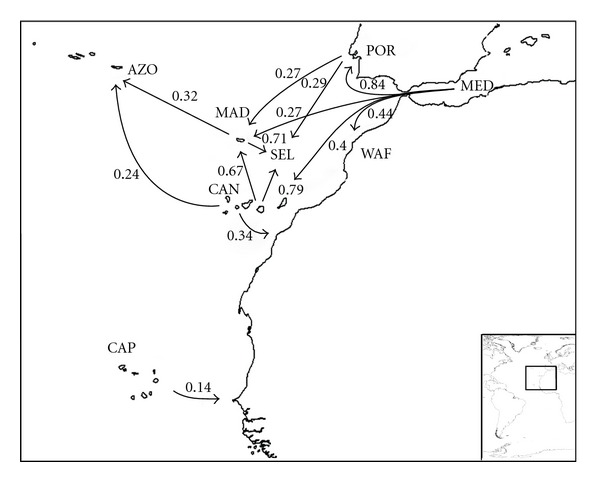
Probable colonization patterns of rissoid fauna in the Macaronesian islands, Northeast-Atlantic, and Mediterranean. The arrows represent the probable main flux direction of faunas, and the associated numbers represent, for each pair of areas, the higher of the two similarity index values computed as described in the methods, abbreviations as in Table 1.

**Figure 7:**
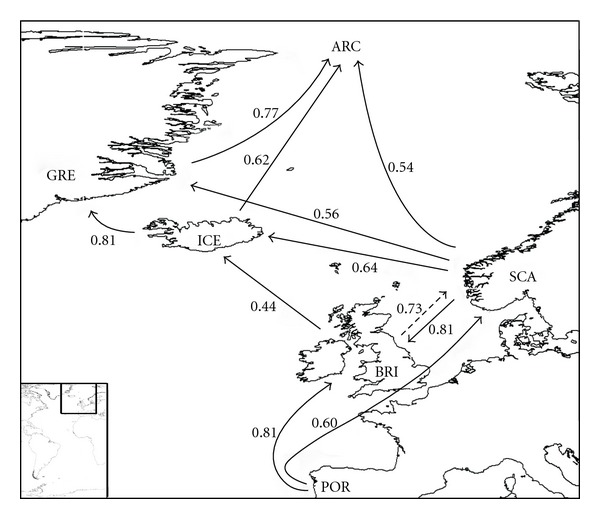
Probable colonization patterns of rissoid fauna in the Northeast Atlantic. The arrows represent the probable main flux direction of faunas, and the associated numbers represent, for each pair of areas, the higher of the two similarity index values computed as described in the methods, abbreviations as in Table 1.

**Figure 8:**
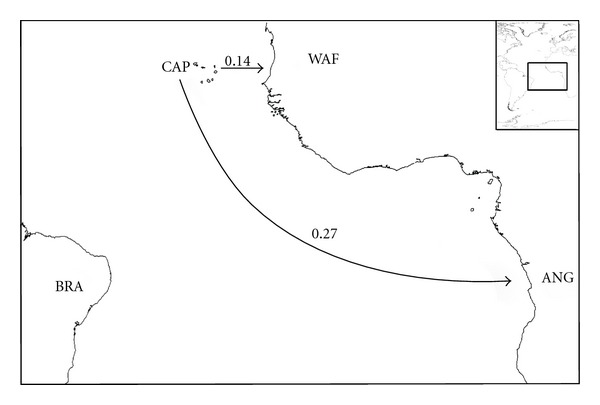
Probable colonization patterns of rissoid fauna in the Central East-Atlantic. The arrows represent the probable main flux direction of faunas, and the associated numbers represent, for each pair of areas, the higher of the two similarity index values computed as described in the methods, abbreviations as in Table 1.

 The results of the analysis of the probable directions of faunal flows (using the *X*_*A*_ and *X*_*B*_ indices; see Section 2) are summarized in Figures 3–8, and support the patterns found by both the PAE analysis and the geographical distribution. Four main source areas for rissoids emerge: Mediterranean, Caribbean, Canaries/Madeira archipelagos, and the Cape Verde archipelago. In the western Atlantic, a rissoid movement originating in the Caribbean seems to have developed southwards to Brazil (Figure 3) and northwards to the Carolinian Province (Figure 4). A southward movement of rissoids, from the Arctic down to the Virginean Province, is envisaged in Figure 4, with a faunal break zone, between the Carolinian and the Virginian Provinces (Figure 4), as well as between Brazil and Southern South America (Figure 5).

In the eastern Atlantic, the patterns are more complicated (Figures 6 and 7). It seems clear that the Mediterranean is the source area for a northwards movement to Portugal, British Isles, and Scandinavia (Figure 7). Scandinavia seems to be the source area for both Iceland and Arctic (64 and 54%, resp.) and Iceland probably played an important role as a source for both Greenland and Arctic. The Mediterranean is weakly related with the West-African shores (44%, Figure 6). The relationships between the Azores and both Madeira and Canaries are weak (32 and 24%, resp.), and Canaries seem to be the main source of the rissoid fauna of Madeira (67%) and Selvagens (79%). Cape Verde archipelago is isolated from all sites (Figure 8), its highest relationship being with Angola (27%).

## 4. Discussion

### 4.1. Geographical Distribution

**Figure 9:**
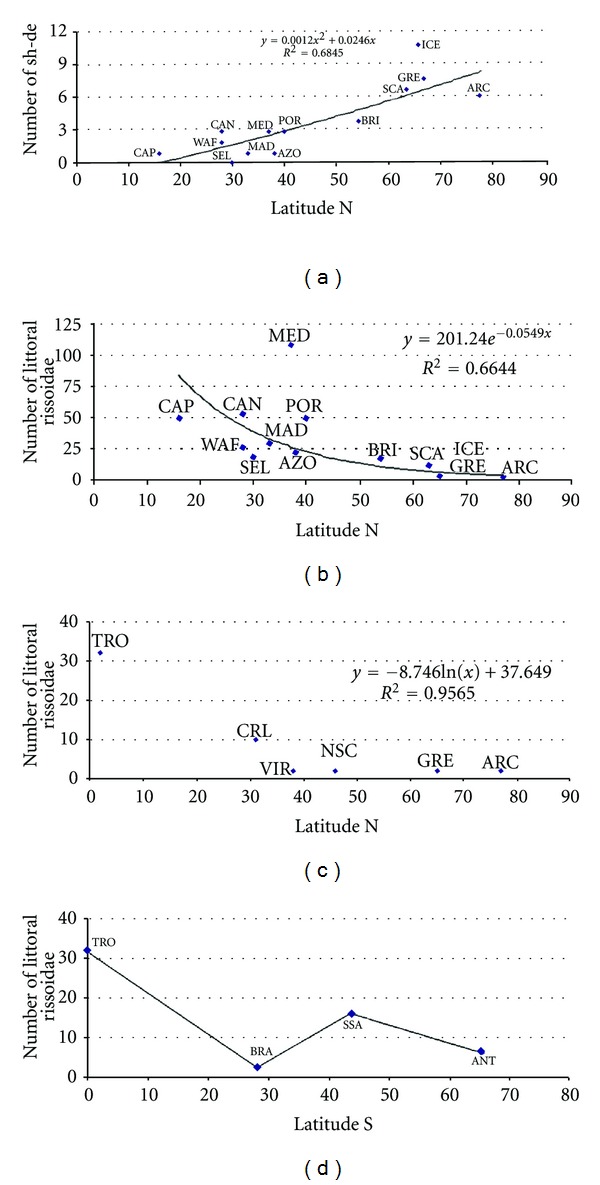
Relation between (a) number of rissoids with large bathymetrical range (#sh-de: shallow-deep) and latitude in the eastern Atlantic; (b) number of littoral rissoids and latitude in the eastern Atlantic; (c) number of littoral rissoids and latitude in the western Atlantic; (d) number of littoral rissoids and latitude in the western Atlantic.

 It is beyond the scope of this paper to discuss in detail all hypotheses related with Rapoport's latitudinal rule (e.g., the seasonal variability hypothesis [2, 4], the differential extinction hypothesis [3], the competition hypothesis [5–7], or the Milankovitch climate oscillations [8]), but one of the corollaries of the seasonal variability hypothesis is that, at low latitudes, the expected bathymetrical range of a given species, in average, should be lower than at high latitudes. Stevens [3] verified this pattern for the Pacific fishes, and a similar pattern is shown by the rissoids of the Norteastern Atlantic (Figure 9(a)).

 Roy et al. [9, 10], who used lists with 3,916 marine Caenogastropod species geographically distributed along the north and Central America shores between 10°S–83°N, confirmed the latitudinal gradient pattern, with the number of species decreasing with latitude. A similar analysis using our Rissoidae database (littoral species only) conforms to the general pattern of latitudinal diversity gradient described in [9, 15, 16, 176, 177] and shows an evident decline of the number of rissoid species with latitude (Figures 9(b)–9(d)). Important asymmetries in the geographical distribution of the mollusc species were found in [12], when they compared the north and south hemispheres of the east-Pacific coast of America. In the northern hemisphere, there is correspondence between the diversity latitudinal gradient and SSTs, but in the southern hemisphere, in particular from 40 to 60°S, the number of species increases with latitude, even though SSTs decrease monotonically with this variable. This pattern is also evident with the shallow Rissoidae along the west-Atlantic coasts of South America (Figures 9(d)) and the explanation is dependent on the coastal area (comprising depths less than 200 m) which, according to Valdovinos et al. [12], is a factor that better explains biodiversity than SSTs. Thus, the increase of the number of shallow rissoids with latitude along the southern South-America shores (Figure 9(d)) is due to the area effect of the Magellan fjords, which played an important role as refugia during glacial periods, locally enhancing the speciation evolutionary processes [12, 178]. 

It is noteworthy to emphasize that the Mediterranean area has more species than expected for similar latitudes (31–43°N) (Figure 9(b)). This is certainly due to the high sampling effort for this region, but we think that other reasons are also behind this fact (see below). A similar trend was also reported in several other taxonomic groups (Hydromedusae, Siphonophora, Chaetognatha, Appendicularia, Salpida, Cephalopoda, Euphausiacea, Decapoda, and Pisces) [15, 179], reinforcing the Mediterranean as an area of high marine biodiversity. This is even more interesting if we think that the Mediterranean area was repopulated just 5.33 Ma ago, when the “Messinian Salinity Crisis” ended [180–182]. This dramatic event occurred between 5.96–5.33 Ma and provoked an almost complete annihilation of the Mediterranean marine fauna and flora [183, 184]. The desiccation of the Mediterranean Sea happened because of the closure of the Rifian corridor, a marine pathway in the northwest of Morocco, which connected the Mediterranean with the Atlantic [185–187]. This caused an impressive drop in sea level, exceeding 1,500 m, and thick evaporitic series deposited in the Mediterranean basin [188]. The reopening of the connection between the Atlantic and the Mediterranean happened 5.33 Ma ago and, although there are different hypothesis under discussion such as tectonic movements of the crust [189], the most plausible explanation for the reflooding of the Mediterranean Sea is the retrogressive erosion in the Gibraltar strait [190]. The marine molluscs that recolonized the Mediterranean basins were the remnants of an impoverished Miocene Lusitano-Atlantic fauna [184], and contemporaneous of the “Messinian Salinity Crisis,” lacking several Tethyan relics [191]. This malacofauna was severely impacted by climatic changes—the decrease of the SSTs, increase of the ice volume at Antarctic to more than 50% than the present one [192], and the lowering of the mean sea level in about 40 m [184]. The Pliocene and Pleistocene glacial cycles heavily affected the eastern Atlantic shores, but the “buffer” zone provided by the Mediterranean and acted as a refugia zone, especially in the southern shores [193], and this may be the reason for the high number of rissoid species that this area possesses nowadays.

 By contrast, the low number of rissoids on the Virginian Province (only 7 species) is probably related with the predominance of sandy bottoms on the littoral of this biogeographical Province, and with the multiple lagunar and estuarine systems, which are inhospitable to the benthic algae where many species of these micromolluscs live [194]. Van Reine et al. [195] provide a similar explanation to explain the poverty of the west-African tropical algal flora. This happens because suitable hard surfaces for seaweed attachment are scarce, with large areas of sand and mangrove, the wave-exposure is high (very few sheltered sites occur) and also because the inshore salinity is often reduced. This has a profound impact on the epibenthonic malacofauna that lives associated with these algae, and the scarcity of algae by the reasons previously discussed is the most likely explanation for the low number of the west-African rissoids.

### 4.2. Endemic Species

 By definition, “a species can be endemic to an area for two different reasons: (a) because it has originated in that place and never dispersed, or (b) because it now survives in only a part of its former wider range” [196]. We do not know any endemic rissoid to the Azores, Madeira, or Canaries that is documented in the fossil record as formerly having a broader geographic distribution [197, 198]. So, they are autochthonous descendents of immigrants, rather than geographic relicts.

 In some areas, a few genera went through a speciation process that led to a high number of both species and endemics, for example, *Alvania*, *Crisilla*, *Onoba*, *Pusillina*, *Rissoa,* and *Setia* at the Mediterranean; *Benthonellania* and *Rissoina* at the Caribbean; *Manzonia* and *Crisilla* at the Madeira, Selvagens, and Canaries archipelagos; *Crisilla* and *Schwartziella* at the Cape Verde archipelago; *Onoba* at Iceland; and *Cingula* at the geographically isolated Saint Helena Island.

 Mironov [199] proposed the concept of “centers of marine fauna redistribution” as a dynamic concept that accommodates an evolutionary perspective for a geographical area, integrating two usually opposite concepts: the center of origin, and the center of accumulation. He emphasized that a center of redistribution should be regarded as a biogeographic unit with three developmental stages, succeeding each other, in time: first, it is a stage of accumulation of species, it then evolves to a stage of speciation and, the last phase, is the dispersal stage. Thus, such concept is the “consecutive stage of development of an integrated and complicated event,” uniting the opposing accumulation and dispersal concepts in a given area, which is designated as a “center of redistribution” [196]. According to these authors, in the past, during the Cretaceous and the Palaeogene, the Mediterranean part of the Tethys Sea acted as a center of origin, but it lost the role of a centre of speciation and dispersal during the Miocene and Pliocene. However, our data show that the high number of rissoids (160 species), as well as the high number of endemics (71) that are reported to the Mediterranean area, must be related to the first two stages proposed by Mironov [199]—accumulation and speciation—which are certainly related to the protective role of the Mediterranean as a refugee during glacial episodes. In the present, the other center of speciation in the Atlantic is the Caribbean area, which is described by Krylova [196] as a center of redistribution, and considered by Briggs [200] as a centre of origin.

 We must stress the high percentage of endemics that occur in the isolated islands of Saint Helena, Tristan da Cunha, and at Cape Verde archipelago (more than 85% of endemics), and also at the Azores (44.7%) thus reinforcing the legislative protective actions that the local governments have implemented in these islands during the recent years. The Cape Verde islands probably received the first rissoids from West-African shores, from where it distances nowadays just about 500 km but must have undergone a long period of isolation, which explains such a high number of endemics. Also, our results (both PAE and *X_A_* and* X_B_* indices) point to a faunal breakdown zone between Cape Verde archipelago and the nearest areas, with very weak relationships with the Canary Islands (7%) and West-African shores (14%), and a little bit higher similarities with Angola (27%).

### 4.3. Bathymetrical Zonation and Modes of Larval Development

 It is a well-known fact that biotic communities in high latitudes are usually rich in nonplanktotrophic species [157, 201]. This is Thorson's rule “pelagic development reveals a clear biological polarity: from low towards high latitude pelagic development disappears progressively and becomes replaced by direct development, demersal development, and viviparity” [202, 203]. Thorson's original formulation related also pelagic development with depth, saying that the number of species with such a type of development would gradually diminish from the shallow shelf downwards to the abyssal depths, until its complete disappearance [202] a concept that did not hold [204, 205].

 It is interesting that the few planktotrophic deep rissoid species are indeed those with higher density and with wider geographical ranges (e.g., *Obtusella intersecta *and *Benthonella tenella *[27]. Rex and Warén [206] provided a plausible explanation for this. These authors noted that the higher predominance of planktotrophic carnivore species among the Meso and Neogastropoda with increasing depths, a phenomenon detected by Grahame and Branch [207], may be due to the enhancement of this mode of development at bathyal depths, because of the advantages that the larval dispersal confers in an environment where resources are unevenly distributed. 

 The first fossil record of the Rissoidae family is from the lower Jurassic of the Tethys Sea [21, 208] and the oldest records in Europe are from the Toarcian of Italy [209] and from the Bajocian of England [210]. Some modern rissoid genera (e.g., *Rissoa* and *Alvania*) are known since the Early Miocene (Eggenburgian) of the Central Paratethys [211]. The protoconch morphologies of Middle Miocene (Badenian) rissoids belonging to modern genera (*Rissoa*, *Manzonia*, and *Alvania*) are similar to their extant Mediterranean and NE Atlantic congeners, and, interestingly, all of these species possess a planktotrophic mode of larval development [211]. 

The ancestral of the Rissoidae presumably had a planktotrophic mode of development [208]. Jablonski [212] stated that species with a planktotrophic larval development and long-distance dispersal usually have wider geographical ranges, longer geological ranges, and smaller speciation and extinction rates than nonplanktotrophic relatives. He detected changes in the modes of larval development of Cretacian molluscs, from planktotrophic to nonplanktotrophic, but the reverse was not found. In the Mediterranean, sibling species were found, almost identical in their teleoconchs and differing mostly in their protoconchs: one multispiral, denoting a planktotrophic larval development, the other one paucispiral, denoting a nonplanktotrophic larval development [213]. This unidirectional change in the mode of larval development may also explain the high percentage of nonplanktotrophic rissoid species that are present in the Atlantic archipelagos/islands (Tables 6 and 7). Moore [214] at Rockall, an isolated and inhabited islet located in the north Atlantic (57°N, 13°W), first noted this phenomenon and stated that a pelagic phase may be an advantage for dispersal, but it may exclude species from certain habitats, namely, from oceanic islands. Moreover, larvae of nonplanktotrophic species are protected from the surrounding environment during the initial phase of their development, by the walls of the egg. In addition, they do not need external food supply, as vitelum provides them all energy they need to account for the completion of the metamorphosis [207]. Last but not the least, when the juveniles emerge from the egg, they are inserted in their natural habitat, not incurring into the risks that adversely affect the larvae of planktotrophic species, which may settle into nonsuitable substrates [204]. Thus, it is not such a surprise that at high latitudes, where the abiotic conditions are more extreme for shallow species (low salinity, low temperatures during a large period of the year) [215] and resources for larval stages are available only during short periods, there exists such a predominance of nonplanktotrophic rissoid species. 

In the absence of a phylogenetic analyses for this family, one can only speculate that during geological times, the ancestral(s) (either planktotrophic or nonplanktotrophic) dispersed, reached an oceanic island by natural means (see [198, 216] for a review), and was able to establish there a viable population. Once in the island, there are evolutive advantages in diminishing the dispersal capabilities [217, 218]. If the ancestral was a planktotrophic species, these selective forces may induce the change to a nonplanktotrophic mode of development. In doing so, this automatically reduces the chances of the larvae to be lost into the open ocean. This may be achieved by two ways: (i) reduction of the period of dispersion, or (ii) by larval behaviour related with the selection of favourable marine currents that keep the larvae nearby the substrates that are appropriated to the adult benthonic life [219]. In the first case, the change of larval development produces a new species, with identical teleoconch (or very similar) to the ancestral, but with a different protoconch. This geologically “instantaneous” speciation event, without intermediary forms, is common in both the fossil record and among the extant caenogastropods, of which several pairs of sibling species are reported [47, 213, 220–224]. Speciation may also occur from ancestral planktotrophic species by means of the disruption of a formerly wider geographical range (e.g., due to the glacial-interglacial cycles). Gofas [116] reported four pairs of such rissoid species from West African shores, all of them with planktotrophic development and closely related to European congenerics: *Alvania africana*/*A*. *beani*, *Alvania marioi*/*A*. *cancellata*, *Alvania gofasi*/*A*. *zetlandica*, and *Crisilla transitoria*/*C*. *semistriata*.

As it is not possible to invert the loss of the planktotrophic phase, in a relatively short interval, the planktotrophic ancestral may originate one or several species, by adaptive radiation, each species occupying a different niche. This promotes the increase of the number of nonplanktotrophic species, usually with a restricted range of dispersion (Strahtmann, 1986). Several examples are known from oceanic islands that elucidate the above-mentioned mechanisms: at Madeira, Selvagens, and Canary Islands, the rissoid genera *Alvania*, *Crisilla*,* Manzonia*, and *Rissoa *produced a high number of species, whereas at Cape Verde, the most specious genera were *Alvania*, *Crisilla*, and *Schwartziella*. In the Azores, *Alvania* and *Setia *are also good examples (Table 1). Similar speciation events also happened in other families of gastropods (e.g., in Cape Verde, Conidae, with about 45 species/subspecies, and *Euthria*, with 20 species) [225, 226].

 Currently, we are unable to choose between the following hypotheses:

1. colonization by an ancestral (either planktotrophic or nonplanktotrophic), followed by speciation with adaptive radiation;
2. several independent colonizations, spaced in timed, by an ancestral that originates a different species, without adaptive radiation;
3. both hypotheses above described.

### 4.4. Biotic Similarities between Areas: Parsimony Analysis of Endemicity and Probable Directions of Faunal Flows

Although many authors postulate a stepping-stone dispersal through the chain of seamounts located between Portugal and Madeira archipelago (Gorringe, Josèphine, Ampère, and Seine), especially during the sea level low stands associated with the Pleistocene glacial periods [88, 167, 198], our analysis shows that the rissoid relationships between these areas are weak (Figure 6). This methodology does not provide a clear migratory route from the Mediterranean, Portuguese shores, or West-African shores towards the Macaronesian archipelagos (“*sensu strictum*”: Azores, Madeira, Selvages, and Canaries), all computed pairs of areas show low relationships (POR-MAD = 27%; POR-SEL = 29%; MED-MAD = 27%; MED-CAN = 40%; MAD-WAF = 44%; CAN-WAF = 34%, Figure 6). Interestingly, Canaries act as a probable source of rissoid fauna, instead of recipient, when compared to all areas, with the sole exception of the Mediterranean.

Another interesting feature is the isolation of the Cape Verde archipelago, which is very weakly related with the “*sensu strictum*” Macaronesian archipelagos (Azores, Madeira, Selvagens, and Canaries archipelagos, Figure 8). This reinforces the idea that, at least for the marine species, Cape Verde should not be included in the Macaronesian area, as already suggested by a number of authors [167, 227, 228].

## Figures and Tables

**Table 1:** Number of Rissoid species, by genus in the Atlantic Ocean and in the Mediterranean Sea. ARC: Arctic; GRE: Greenland; ICE: Iceland; SCA: Scandinavia; BRI: British Isles; POR: Portugal; MED: Mediterranean Sea; LUS: Lusitanian seamounts; MET: Meteor seamounts; AZO: Azores; MAD: Madeira, Porto Santo and Desertas; SEL: Selvagens Islands; CAN: Canary Islands; CAP: Cape Verde; WAF: west-African coast; ANG: Angola; NSC: New Scotia biogeographic province; VIR: Virginian biogeographic province; CRL: Carolinian biogeographic province; TRO: Tropical biogeographic province (Caribbean); BRA: Brazil; STH: Santa Helena; TRS: Tristan da Cunha; SSA: southern South-America; ANT: Antarctic.

	ARC	GRE	ICE	SCA	BRI	POR	MED	LUS	MET	AZO	MAD	SEL	CAN	CAP	WAF	ANG	NSC	VIR	CRL	TRO	BRA	STH	TRS	SSA	ANT
*Alvania*	4	7	10	12	10	30	74	10	9	19	22	13	28	25	29	5	1	1	2	21	1			1	
*Amphirissoa*									1	1										1					
*Benthonella*			1	1	1	1	1	1	1	1	1		1				1	1	1	1					
*Benthonellania*								1		1	2				3	1			4	5	2	1			
*Boreocingula*	2	2	1	1	1																				
*Botryphallus*						1	1			1	1	1	1	1											
*Cingula*	1			1	1	1				1	1									2		9			
*Crisilla*				1	1	1	7	1		1	8	6	10	6	3	1									
*Folinia*																				2					
*Frigidoalvania*	3	2	1	1													3	2							
*Galeodinopsis*														1	1	1									
*Gofasia*						2		5	2		1	1	1												
*Lironoba*																				1					
*Manzonia*				1	1	2	1	6		1	7	7	10		2					1					
*Microstelma*																				4		1			
*Obtusella*			2	2	2	1	2			2	1		1	2	1	1									
*Onoba*	3	5	9	3	2	3	7			1	2	1	5				4	2				2	6	22	7
*Peringiella*						1	2																		
*Plagyostila*						1	1							1	2										
*Pontiturboella*							1																		
*Porosalvania*									8																
*Powellisetia*																							1	3	1
*Pseudosetia*			1	2	1	3	2	1	1	1	1	1	2		1										
*Pusillina*				2	2	7	11	1	2	1	3		5	1	2	1	1	1						1	
*Rissoa*				3	4	12	26	1		2	7	5	15	1	2							2			
*Rissoina*						1	3		1				1	2	1	1			4	18	2				
*Rudolphosetia*						1	1				1	1	1												
*Schwartziella*									1					26	1				4	9	2	5			
*Setia*					1	6	18			5	3	1	6		1					1					
*Simulamerelina*																			1	3					
*Stosicia*																				2					
*Voorwindia*							1																		
*Zebina*							1				2	1	2	1	1				2	6					

Total of Rissoidae species	13	16	25	30	27	74	160	27	26	38	63	38	89	67	50	11	10	7	18	77	7	20	7	27	8

**Table 2:** Number of endemic Rissoidae, other abbreviations as in Table 1.

	ARC	GRE	ICE	SCA	BRI	POR	MED	LUS	MET	AZO	MAD	SEL	CAN	CAP	WAF	ANG	NSC	VIR	CRL	TRO	BRA	STH	TRS	SSA	ANT
*Alvania*			2			1	37	1	7	10	3	2	7	20	5				1	19				1	
*Amphirissoa*																									
*Benthonella*																									
*Benthonellania*															1				1	2	1				
*Boreocingula*	1	1																							
*Botryphallus*										1				1											
*Cingula*	1																			2		9			
*Crisilla*							3	1			1		1	6	1										
*Folinia*																				2					
*Frigidoalvania*																									
*Galeodinopsis*																									
*Gofasia*								3	2																
*Lironoba*																				1					
*Manzonia*								5		1	2	1	4		1					1					
*Microstelma*																				4		1			
*Obtusella*							1			1				1											
*Onoba*			1			2	6			1			1									2	6	14	2
*Peringiella*							1																		
*Plagyostila*															1										
*Pontiturboella*																									
*Porosalvania*									8																
*Powellisetia*																								2	1
*Pseudosetia*				1																					
*Pusillina*							3		1		1			1										1	
*Rissoa*							11						3	1								2			
*Rissoina*									1					1					1	13					
*Schwartziella*									1					26	1					5		4			
*Setia*							9			3	1		1		1										
*Simulamerelina*																				2					
*Stosicia*																				2					
*Voorwindia*																									
*Zebina*														1	1					4					

Total of endemic Rissoidae	2	1	3	1	0	3	71	10	20	17	8	3	17	58	12	0	0	0	3	57	1	18	6	18	3
Total of Rissoidae species	13	16	25	30	27	74	160	27	26	38	63	38	89	67	50	11	10	7	18	77	7	20	7	27	8
*% endemics*	15.4	6.3	12.0	3.3	0.0	4.0	44.4	37.0	76.9	44.7	12.7	7.9	19.1	86.6	24.0	0.0	0.0	0.0	16.7	74.0	14.3	90.0	85.7	66.7	37.5

**Table 3:** Number of shared Rissoidae species, other abbreviations as in Table 1.

	ARC	GRE	ICE	SCA	BRI	POR	MED	LUS	MET	AZO	MAD	SEL	CAN	CAP	WAF	ANG	NSC	VIR	CRL	TRO	BRA	STH	TRS	SSA	ANT
*ARC*	13																								
*GRE*	10	16																							
*ICE*	8	13	25																						
*SCA*	7	9	16	30																					
*BRI*	2	4	11	22	27																				
*POR*	0	1	7	18	22	74																			
*MED*	0	0	6	17	21	62	160																		
*LUS*	0	0	2	5	6	11	9	27																	
*MET*	0	0	1	1	1	1	1	2	26																
*AZO*	0	0	3	5	6	8	7	5	5	38															
*MAD*	0	0	4	5	8	17	17	7	1	12	63														
*SEL*	0	0	0	0	1	11	11	4	0	3	27	38													
*CAN*	1	2	7	14	17	32	36	7	2	9	42	30	89												
*CAP*	1	0	2	3	4	5	5	2	0	3	4	1	4	67											
*WAF*	0	0	3	7	9	18	22	5	0	4	12	6	17	7	50										
*ANG*	0	0	1	2	2	3	3	0	0	2	2	0	2	3	11	11									
*NSC*	4	5	6	2	2	1	1	1	1	1	1	0	2	0	0	0	10								
*VIR*	2	3	4	2	2	1	1	1	1	1	1	0	2	0	0	0	7	7							
*CRL*	0	0	1	1	1	1	2	1	1	1	2	0	2	0	0	0	1	1	18						
*TRO*	0	0	1	1	1	2	3	1	2	2	3	0	3	0	1	0	1	1	15	77					
*BRA*	0	0	0	0	0	0	0	0	0	0	0	0	0	0	0	0	0	0	3	5	7				
*STH*	0	0	0	0	0	0	0	0	0	0	1	0	0	0	1	0	0	0	1	1	1	20			
*TRS*	0	0	0	0	0	0	0	0	0	0	0	0	0	0	0	0	0	0	0	0	0	0	7		
*SSA*	0	0	0	0	0	0	0	0	0	0	0	0	0	0	0	0	0	0	0	0	0	0	0	27	
*ANT*	0	0	0	0	0	0	0	0	0	0	0	0	0	0	0	0	0	0	0	0	0	0	0	4	8

**Table:** (a)

	ARC		GRE		ICE		SCA		BRI		POR		MED		LUS		MET		AZO		MAD		SEL		CAN		CAP	
	lit	deep	lit	deep	lit	deep	lit	deep	lit	deep	lit	deep	lit	deep	lit	deep	lit	deep	lit	deep	lit	deep	lit	deep	lit	deep	lit	deep
*Alvania*		2		4		7	2	8	5	5	17	13	50	20		9		6	11	8	13	9	10	2	17	11	17	8
*Amphirissoa*																1				1								
*Benthonella*						1		1		1		1		1		1		1		1		1				1		
*Benthonellania*																		1		1		2						
*Boreocingula*	1	1	1		1		1	1	1	1																		
*Botryphalus*											1		1						1		1		1		1		1	
*Cingula*		1					1		1		1								1		1							
*Crisilla*							1		1		1		6					1	1		7		6		9	1	6	
*Folinia*																												
*Frigidoalvania*		1																										
*Galeodinopsis*																											1	
*Gofasia*												2				2		5				1		1		1		
*Lironoba*																												
*Manzonia*							1		1		2		1					5	1		6		7	1	10			
*Microstelma*																												
*Obtusella*														1						1							1	
*Onoba*	1		1	2	1	3	1	1	1		2		3	3					1		1	1	1		3	1		
*Peringiella*											1		2															
*Plagyostila*											1		1														1	
*Pontiturboella*																												
*Porosalvania*																8												
*Powellisetia*																												
*Pseudosetia*						2		2		1		3		2		1		1		1		1		1		2		
*Pusillina*							1		1		5	1	9			2		1	1		1	1			2	2		1
*Rissoa*							3		5		12		23						2		7		5		14		1	
*Rissoina*											1		2			1									1		2	
*Rudolphosetia*											1		1								1		1		1			
*Schwartziella*																1											20	6
*Setia*									1		6		17						5		3		1		6			
*Simulamerelina*																												
*Stosicia*																												
*Voorwindia*													1															
*Zebina*													1								2		1		2			1

Total number of Rissoidae	2	5	2	6	2	12	11	13	17	8	51	20	118	28	0	26	0	21	24	13	43	16	33	5	66	19	50	16

**Table:** (b)

	WAF		ANG		TRS		STH		NSC		VIR		CRL		TRO		BRA		SSA		ANT	
	lit	deep	lit	deep	lit	deep	lit	deep	lit	deep	lit	deep	lit	deep	lit	deep	lit	deep	lit	deep	lit	deep
*Alvania*	18	11	6											1	10	3				1		
*Amphirissoa*																1						
*Benthonella*										1		1		1		1						
*Benthonellania*		3		1				1						3		4		2				
*Boreocingula*																						
*Botryphallus*																						
*Cingula*							4									2						
*Crisilla*	1	2	1																			
*Folinia*															1							
*Frigidoalvania*									1	1	1	1										
*Galeodinopsis*	1																					
*Gofasia*																						
*Lironoba*																						
*Manzonia*	1	1													1							
*Microstelma*							1									3						
*Obtusella*																						
*Onoba*					2	4	1		1	1	1	1							14	4	5	
*Peringiella*																						
*Plagyostila*	2																					
*Pontiturboella*																						
*Porosalvania*																						
*Powellisetia*						1													3		1	
*Pseudosetia*		1																				
*Pusillina*	1	1								1		1							1			
*Rissoa*	1						2															
*Rissoina*	1		1										4		13							
*Rudolphosetia*																						
*Schwartziella*	1						1						4		8		2					
*Setia*	1															1						
*Simulamerelina*													1		3							
*Stosicia*															2							
*Voorwindia*																						
*Zebina*	1												2		4							

Total number of Rissoidae	29	19	8	1	2	5	9	1	2	4	2	4	11	5	42	15	2	2	18	5	6	0

**Table 5:** Mode of larval development of the Rissoidae: np: nonplanktotrophic species; p: planktotrophic species, other abbreviations as in Table 1.

		*Alvania*	*Amphirissoa*	*Benthonella*	*Benthonellania*	*Boreocingula*	*Botryphallus*	*Cingula*	*Crisilla*	*Folinia*	*Frigidoalvania*	*Galeodinospis*	*Gofasia*	*Lironoba*	*Manzonia*	*Microstelma*	*Obtusella*	*Onoba*	*Peringiella*	*Plagyostila*	*Pontiturboella*	*Porosalvania*	*Powellisetia*	*Pseudosetia*	*Pusillina*	*Rissoa*	*Rissoina*	*Rudolphosetia*	*Schwartziella*	*Setia*	*Simulamerelina*	*Stosicia*	* Voorwindia*	*Zebina*	Total number of rissoids
ARC	np	4				2		1			3							3																	13
	p																																		0
GRE	np	7				2					2							5																	16
	p																																		0
ICE	np	9				1					1							8						1											20
	p	1		1													1	1																	4
SCA	np	7				1		1			1							2						2	1										15
	p	5		1					1						1		1	1							2	2									14
BRI	np	3				1		1										1						1	1					1					9
	p	7		1					1						1		1	1							2	3									17
POR	np	17					1	1					2		1			2						3	2	3		1		6					39
	p	13		1					1						1		1	1	1	1					5	9	1								35
MED	np	51					1		2									4						2	5	10		1		17					93
	p	17		1					3						1		2	1	1	1					6	12	1						1	1	48
LUS	np	5			1				1				5		6									1	1										20
	p	5		1																						1									7
MET	np	9	1										2									8		1	2		1		1						25
	p			1																															1
AZO	np	17	1		1		1	1	1						1		1	1						1		2				5					33
	p	2		1													1								1										5
MAD	np	20			1		1	1	3				1		7			2						1	2	6		1		3				1	50
	p	2		1	1												1								1	1								1	8
SEL	np	12					1		2				1		7			1						1		4		1		1				1	32
	p	1																								1									2
CAN	np	23					1		3				1		10			4						2	2	8		1		4				1	60
	p	5		1					2								1	1							3	6	1							1	21
CAP	np	20					1		6																1	1	1		26					1	57
	p	5										1					2			1							1								10
WAF	np	18			2				2						2									1	1				1	1				1	29
	p	11			1				1			1					1			2					2	1	1								21
ANG	np	2																																	2
	p	3			1				1			1					1								1		1								9
NSC	np	1									3							4							1										9
	p			1																															1
VIR	np	1									2							2							1										6
	p			1																															1
CRL	np	1																									1		1		1			1	5
	p	1		1																							2		3					1	8
TRO	np	7	1											1													7		4		3	1		3	27
	p	1		1						1																	7		3			1		2	16
BRA	np																										1								1
	p	1																									1		2						4
STH	np				1			8																											9
	p							1																					1						2
TRS	np																	1																	1
	p																																		0
SSA	np																	18					2		1										21
	p																																		0
ANT	np																	6					1												7
	p																																		0

**Table 6:** Number of littoral Rissoidae with nonplanktotrophic mode of larval development, other abbreviations as in Table 1.

	ARC	GRE	ICE	SCA	BRI	POR	MED	AZO	MAD	SEL	CAN	CAP	WAF	ANG	NSC	VIR	CRL	TRO	BRA	STH	TRS	SSA	ANT
*Alvania*						8	36	10	12	9	14	15	10	2				5					
*Amphirissoa*																							
*Benthonella*																							
*Benthonellania*																							
*Boreocingula*	2	1	1	1	1																		
*Botryphallus*						1	1	1	1	1	1	1											
*Cingula*				1	1	1		1	1											3			
*Crisilla*							2	1	3	2	2	6											
*Folinia*																							
*Frigidoalvania*															1	1							
*Gofasia*																							
*Lironoba*																							
*Manzonia*						1		1	6	7	10		1										
*Microstelma*																							
*Obtusella*																							
*Onoba*	1	1	1	1	1	2	3	1	1	1	3				1	1						13	5
*Peringiella*																							
*Plagyostila*																							
*Powellisetia*																						2	1
*Pseudosetia*																							
*Pusillina*				1	1	1	4															1	
*Rissoa*						3	10	2	6	4	8	1											
*Rissoina*												1					1	5					
*Rudolphosetia*						1	1		1	1	1												
*Schwartziella*												20	1				1	4					
*Setia*					1	6	17	5	3	1	4		1										
*Simulamerelina*																	1	3					
*Stosicia*																		1					
*Voorwindia*																							
*Zebina*									1	1	1		1				1	2					

Total number of Rissoidae	3	2	2	4	5	24	74	21	35	27	44	44	14	2	2	2	4	20	0	3	0	16	6

**Table 7:** Number of littoral Rissoidae with planktotrophic mode of larval development, other abbreviations as in Table 1.

	ARC	GRE	ICE	SCA	BRI	POR	MED	LUS	MET	AZO	MAD	SEL	CAN	CAP	WAF	ANG	NSC	VIR	CRL	TRO	BRA	STH	TRS	SSA	ANT
*Alvania*				2	5	9	12	3		1	1	1	3	2	8	3									
*Amphirissoa*																									
*Benthonella*																									
*Benthonellania*																									
*Boreocingula*																									
*Botryphallus*																									
*Cingula*																						1			
*Crisilla*				1	1	1	3						2		1	1									
*Folinia*																				1					
*Frigidoalvania*																									
*Galeodinopsis*														1	1	1									
*Gofasia*																									
*Lironoba*																									
*Manzonia*				1	1	1	1																		
*Microstelma*																									
*Obtusella*														1											
*Onoba*																									
*Peringiella*						1	1																		
*Plagyostila*						1	1							1	2										
*Pontiturboella*																									
*Porosalvania*																									
*Powellisetia*																									
*Pseudosetia*																									
*Pusillina*				1	1	4	5				1		2		1										
*Rissoa*				2	3	9	12	1			1	1	6		1										
*Rissoina*						1	1						1	1	1	1			2	5					
*Rudolphosetia*																									
*Schwartziella*																			3	3	2	1			
*Setia*																									
*Simulamerelina*																									
*Stosicia*																				1					
*Voorwindia*							1																		
*Zebina*							1				1		1						1	2					

Total number of Rissoidae	0	0	0	7	11	27	38	4	0	1	4	2	15	6	15	6	0	0	6	12	2	2	0	0	0

**Table 8:** Number of deep Rissoidae with nonplanktotrophic mode of larval development, other abbreviations as in Table 1.

	ARC	GRE	ICE	SCA	BRI	POR	MED	LUS	MET	AZO	MAD	SEL	CAN	CAP	WAF	ANG	NSC	VIR	CRL	TRO	BRA	STH	TRS	SSA	ANT
*Alvania*	2	4	6	5	3	9	14	4	9	7	8	3	9	5	8				1	1					
*Amphirissoa*									1	1										1					
*Benthonella*																									
*Benthonellania*								1		1	1				2							1			
*Boreocingula*																									
*Botryphallus*																									
*Cingula*	1																								
*Crisilla*								1					1		2										
*Folinia*																									
*Frigidoalvania*	1																1	1							
*Galeodinopsis*																									
*Gofasia*						2		5	2		1	1	1												
*Lironoba*																									
*Manzonia*								5							1										
*Microstelma*																									
*Obtusella*										1															
*Onoba*		2	3	1			1				1		1				1	1					1	1	
*Peringiella*																									
*Plagyostila*									8																
*Pontiturboella*																									
*Porosalvania*																									
*Powellisetia*																									
*Pseudosetia*			1	2	1	3	2	1	1	1	1	1	2		1										
*Pusillina*						1	1	1	2		1		2	1	1		1	1							
*Rissoa*																									
*Rissoina*									1																
*Rudolphosetia*																									
*Schwartziella*														6											
*Setia*																									
*Simulamerelina*																									
*Stosicia*																									
*Voorwindia*																									
*Zebina*									1					1											

Total number of Rissoidae	4	6	10	8	4	15	18	18	25	11	13	5	16	13	15	0	3	3	1	2	0	1	1	1	0

**Table 9:** Number of deep Rissoidae with planktotrophic mode of larval development, other abbreviations as in Table 1.

	ARC	GRE	ICE	SCA	BRI	POR	MED	LUS	MET	AZO	MAD	SEL	CAN	CAP	WAF	ANG	NSC	VIR	CRL	TRO	BRA	STH	TRS	SSA	ANT
*Alvania*			1	3	2	4	5	2		1	1		2	3	3										
*Amphirissoa*																									
*Benthonella*			1	1	1	1	1	1	1	1	1		1				1	1	1	1					
*Benthonellania*											1				1	1									
*Boreocingula*																									
*Botryphallus*																									
*Cingula*																									
*Crisilla*																									
*Folinia*																									
*Frigidoalvania*																									
*Galeodinopsis*																									
*Gofasia*																									
*Lironoba*																									
*Manzonia*																									
*Microstelma*																									
*Obtusella*							1																		
*Onoba*																									
*Peringiella*																									
*Plagyostila*																									
*Pontiturboella*																									
*Porosalvania*																									
*Powellisetia*																									
*Pseudosetia*																									
*Pusillina*																									
*Rissoa*																									
*Rissoina*																									
*Rudolphosetia*																									
*Schwartziella*																									
*Setia*																									
*Simulamerelina*																									
*Stosicia*																									
*Voorwindia*																									
*Zebina*																									

Total number of Rissoidae	0	0	2	4	3	5	7	3	1	2	3	0	3	3	4	1	1	1	1	1	0	0	0	0	0
